# Digital Teaching and Learning Media for Nursing and Health Care Courses in Germany: Protocol for a Scoping Review

**DOI:** 10.2196/60427

**Published:** 2025-01-17

**Authors:** Jann Niklas Vogel, Jaqueline Letzin, Stefan Schmidt

**Affiliations:** 1 Faculty of Health, Nursing, Management University of Applied Sciences Neubrandenburg Neubrandenburg Germany

**Keywords:** digital education, digital learning, digital teaching, e-learning, nursing, health care, digital transformation, digital technology, online learning, distance learning, health care education

## Abstract

**Background:**

In Germany, digital transformation and legal regulations are leading to the need to integrate digital technologies into the nursing profession. In addition, to nursing practice, they are also being incorporated into nursing training. Despite comprehensive regulations regarding the use of digital teaching and learning media in nursing education, their specific applicability and implementation vary.

**Objective:**

This study aims to map evidence and identify the main concepts, theories, sources, and knowledge gaps in the use of digital teaching and learning formats in nursing and health care education in Germany.

**Methods:**

The study is planned as a scoping review. The reporting of the study is based on the PRISMA (Preferred Reporting Items for Systematic reviews and Meta-Analyses) 2020 guidelines. The sources of information for the review include six bibliographic databases (MEDLINE via PubMed, Cochrane Library, Web of Science Core Collection, ERIC, PROSPERO, and APA PsycInfo). The search results will be presented in accordance with the PRISMA-ScR (Preferred Reporting Items for Systematic Reviews and Meta-Analyses extension for Scoping Reviews) checklist. The eligibility of studies is based on the population, concept, and context criteria: (1) learners of nursing and health care professions, (2) digital teaching and learning formats, and (3) forms of implementation in Germany since 2007.

**Results:**

The literature search is planned for January 2025. The selection of titles, the coding of the data, and the data analysis are expected to be completed by March 2025.

**Conclusions:**

In Germany, there is a growing interest in integrating digital teaching and learning formats into nursing and health care education. Our scoping review will map applications of digital teaching and learning media in the education of nursing and health care professions in Germany. In this way, the scoping review provides relevant impulses for fields of application and design aspects of digital teaching or learning media for nursing and health care education.

**International Registered Report Identifier (IRRID):**

PRR1-10.2196/60427

## Introduction

### Background

The idea of learning with machines is not new. Back in the 1920s, Sidney L Pressey developed a machine that asked a question and offered four possible answers, like the multiple-choice method [1]. Nowadays, digital technologies are used to create and provide learning materials and to support and control the learning process [2]. In this context, the term “e-learning” describes all forms of learning with electronic or digital media [3]. The introduction of the iPhone in 2007 has been an international milestone in the development of e-learning [3,4]. Mobile devices create the conditions for innovative pedagogical approaches such as flipped classrooms or blended learning, by using modern technologies and various teaching methods to make learning more effective and personalized. In corresponding teaching and learning approaches, the time, place, pace, and scope of learning can be freely chosen [2], giving learners more autonomy and control over their individual learning process. A major advantage of mobile devices is that learners often already possess the necessary technical equipment [5]. The digitalization of nursing and health care education is considered to have particular potential due to the complexity of professional requirements and the constant further development of subject-specific knowledge [6,7]. In addition to the relevance of digital teaching and learning formats in nursing and health care education, there is also a need in Germany to incorporate digitalization into health care professional education. This arises from legal regulations such as § 8 Abs. 8 SGB XI or the E-Health Act, which are intended to promote the digitalization of professional practice and require corresponding skills on the part of nursing professionals. The digitalization of health care professional practice is intended to improve the quality of care and effectively prepare professionals for highly complex care situations, which should ultimately lead to better care outcomes for patients [8-10]. In addition, the topic of digitalization can be found in the new legal foundations of the Act on the Nursing Professions (Pflegeberufegesetz, PflBG), the Training and Examination Regulations for the Nursing Professions (Ausbildungs- und Prüfungsverordnung für die Pflegeberufe, PflAPrV) and the framework curricula and framework training plans. These foundations list forms of digital support for various parts of the nursing process.In this regard, there is a need to develop and integrate digital teaching and learning formats for nursing and health care education [6,11].

### Objectives

In Germany, there are increasing efforts to integrate digital teaching and learning formats into nursing and health care education. This fact leads to the question, for which educational content and didactic concepts digital teaching and learning formats are used in nursing and health care education in Germany? Furthermore, which scientific findings are present about the use of digital teaching and learning formats in nursing and health care education in Germany? Thus, our main objectives are (1) digital teaching and learning formats and (2) forms of implementation in the context of learners in the nursing and health care professions.

## Methods

### Study Design

We conduct a scoping review [12,13] in consideration of published primary sources and reviews. All study designs with the exception of opinion studies, commentaries, editorials, letters to the editor, and pure case reports are considered in order to gain a broad overview of the research field. We operationalize the research question by using the population, concept, and context (PCC) scheme [14]. The population includes learners in the nursing and health care professions, the concept focuses on digital teaching and learning formats, and the context refers to forms of implementation in Germany since 2007. Study reporting is based on the PRISMA-ScR (Preferred Reporting Items for Systematic reviews and Meta-Analyses extension for Scoping Reviews) [15]. The PRISMA-ScR checklist will be available once the review is complete.

### Patient and Public Involvement

Patients and the public are not involved in the design of this protocol. Thus, ethics approval is not required for the scoping review.

### Eligibility Criteria

The eligibility of studies is based on the PCC criteria: (1) learners of nursing and health care professions, (2) digital teaching and learning formats, and (3) forms of implementation in Germany since 2007. The scoping review aims to map evidence and identify the main concepts, theories, sources, and knowledge gaps in the use of digital teaching or learning formats in nursing and health care education in Germany. The electronic search is conducted in German and English. Only titles, which are published since 2007 will be included because the iPhone was released for the first time in 2007, which enables new forms of learning and represents an international milestone in the development of e-learning [3,4]. Since this introduction, research into e-learning using mobile devices has increased.

### Information Sources

The information sources for the scoping review will include six bibliographic databases (MEDLINE, Cochrane Library, Web of Science Core Collection, Education Resources Information Center [ERIC], PROSPERO, and APA PsycInfo). The starting point will be the search in MEDLINE via PubMed as the central database for scientific studies in the health professions. The PubMed search supports the generation of suitable mesh terms for the research question and is adapted to the databases Cochrane Library, Web of Science Core Collection, ERIC, PROSPERO, and APA PsycInfo [16]. The Cochrane Library provides access to up-to-date and reliable research, which is further complemented by the PROSPERO database as a repository of systematic reviews. ERIC covers the educational discourse on the question and Web of Science Core Collection bundles the vocational education and health care focal points of the question in a database. These focal points are further supplemented and completed by research via APA PsycInfo, which contains peer-reviewed journals, studies, books, and dissertations on learning and teaching methods in health care, including nursing didactics.

### Search Strategy

**Figure 1:**
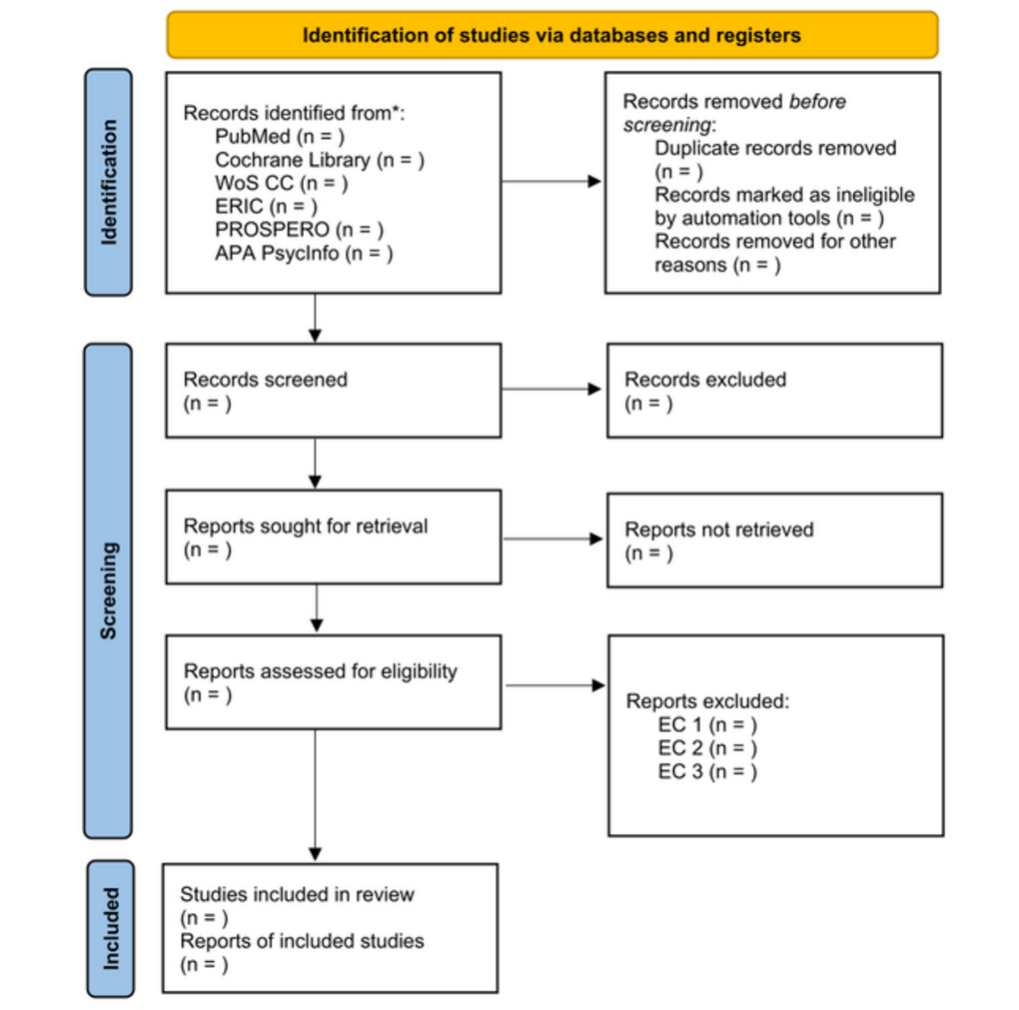
PRISMA-ScR 2020 flow diagram (source: own illustration, adapted from Page et al [17], licensed under CC BY 4.0 [18]). EC: exclusion criteria; EC 1: lack of reference to nursing and health care education; EC 2: no reference to digital teaching and learning formats; EC 3: no reference to the research question. ERIC: Education Resources Information Center; PRISMA-ScR: Preferred Reporting Items for Systematic Reviews extension for Scoping Reviews; PROSPERO: International Prospective Register of Systematic Reviews; WoS CC: Web of Science Core Collection.

The electronic search strategy will be developed iteratively by the team. The search was based on the PRISMA 2020 statement (Figure 1) [17,18]. The search terms and corresponding MeSH (Medical Subject Headings) terms will be derived to address the two main search topics of the scoping review: (1) digital teaching and learning formats and (2) forms of implementation in the context of learners in the nursing and health care professions.

### Selection of Sources of Evidence

Two researchers will search in mentioned databases independently of each other using the search strings and will check all studies for relevance and suitability. After screening, two researchers will compare the search results. Any contradictions or inconsistencies in the study assessment will be clarified through discussion. Studies that met the inclusion criteria will be used for the full-text evaluation. This will also be carried out by two researchers. If necessary, we will contact the authors to obtain further information. The study selection will be concluded with a consensus meeting. Afterward, the data for each included study will be extracted by using a self-developed form in Excel (Microsoft Corp). The results of the literature search will be reported in full once the scoping review is complete. A list of included and excluded studies following full-text screening and individual reasons for exclusion will be reported once the review is complete.

### Data Charting

We will develop an Excel form for coding and recording all data. Two authors code all data independently of each other. The agreement between the codings is calculated using Cohen κ. If necessary, authors will be contacted to obtain further information. A consensus meeting is supposed to conclude the study selection.

### Synthesis of Results

We plan to use qualitative content analysis to form categories in an inductive-deductive alternation [19]. In inductive category formation, categories are derived directly from the material [20]. Deductive category formation defines the categories before the data analysis [20] and refers to the used PCC scheme. This is categorized into setting, digital teaching, and learning format, as well as application formats. The coded data will be summarized in a table and then synthesized using descriptive statistics (relative frequencies).

## Results

The literature search is scheduled for January 2025. We expect to select the relevant studies, code the data, and appraise the studies until March 2025.

## Discussion

### Principal Findings

Preliminary literature searches have shown that various digital technologies are used in nursing and health care education in Germany, including learning programs, learning platforms [6], and virtual reality technologies [21]. Our scoping review will include a detailed list of such applications once the studies have been selected. We will assess the outcomes of such digital technologies in the context of nursing and health care education in Germany. The most interesting aspect of our review will be application areas and requirements, as well as trends in digital nursing and health care education in Germany.

### Comparison to Prior Work

Various digital learning methods have been widely implemented in nursing and health care education. Kimura et al [22] identified interactive online modules and videos as the most frequently used technology-enhanced tools in nursing education. The application period of digital learning formats in nursing and health care training varies [23,24]. Despite the positive associations, the potential of digital teaching and learning is by no means undisputed or proven. For example, the review by Bajpai et al [25] describes a lack of evidence regarding the effectiveness of digital interventions for the training of health care professions. Furthermore, Arkorful and Abaidoo [26] noted potential drawbacks, including reduced interactivity in e-learning environments. Other studies indicated that the achievement of intended learning outcomes through digital teaching or learning media for nursing and health care professions is influenced by variables such as age, training time, and experience of the participants [27,28]. While these findings are valuable, their direct applicability to the German education system is limited, underscoring the importance of examining country-specific implementations of digital education. In this regard, the literature indicates that there are gaps in the implementation of digital media in nursing education [6,29-31]. Our scoping review contributes to this gap by mapping applications of digital teaching and learning media in the education of nursing and health care professions in Germany.

### Strengths and Limitations

This protocol has been rigorously developed and the electronic search syntax was iteratively tested and revised by the authors. Scoping reviews, like this study, contain potential biases in the selection of criteria, the search methodology, and the data analysis. Just German and English-language publications in selected databases will be included. The inclusion of other languages, databases, and additional search terms could lead to an increase in literature sources. Especially regarding the background of the heterogeneous study situation, the methodology of the scoping review is relevant to gain a broad perspective of the use of digital teaching and learning media in nursing and health education in Germany. Nevertheless, the possibility of inaccuracies in a combined interpretation exists.

### Implications for Practice and Dissemination Plan

Findings from the scoping review could be of interest to various stakeholders, including educational institutions, researchers, health professionals, policy makers, and companies developing digital teaching and learning media. Therefore, the results of the review should be published in a peer-reviewed journal.

### Conclusions

Our scoping review maps areas of application of digital teaching and learning methods in nursing and health education in Germany. In addition to the topic areas, the technical implementation methods and the didactic design should also be shown. The findings could be used to establish and expand digital teaching and learning methods in nursing and health care professional educational institutions, especially in Germany.

## Abbreviations

APA PsycInfo: American Psychological Association PsycInfo
ERIC: Education Resources Information Center
MeSH: Medical Subject Headings
PCC: population, concept, and context
PRISMA-ScR: Preferred Reporting Items for Systematic Reviews and Meta-Analyses extension for Scoping Reviews

## Data Availability

This scoping review will be based on previously published data. The datasets generated during or analyzed during this study are available from the corresponding author upon reasonable request.
